# Supplementary material for: Direct formation of copper nanoparticles from atoms at graphitic step edges lowers overpotential and improves selectivity of electrocatalytic CO2 reduction
Source: Commun Chem. 2024 Jun 20;7:140. doi: 10.1038/s42004-024-01218-y (PMC11190262; doi:10.1038/s42004-024-01218-y)
Supplement: Supplementary file 2 — Supplementary Information [file 42004_2024_1218_MOESM2_ESM.pdf]

Direct Formation of Copper Nanoparticles from Atoms at Graphitic Step-Edges Lowers Overpotential and Improves Selectivity of Electrocatalytic CO_2 Reduction

Tom Burwell[1], Madasamy Thangamuthu[1*], Gazi N. Aliev[2], Sadegh Ghaderzadeh[1], Emerson Kohlrausch[1], Yifan Chen,[1] Wolfgang Theis,[2] Luke T. Norman[1], Jesum Alves Fernandes[1], Elena Besley[1], Pete Licence[3], Andrei Khlobystov[1*]

[1]School of Chemistry, University of Nottingham NG7 2RD, UK
[2]School Of Physics & Astronomy, University of Birmingham B15 2TT, UK
[3]School of Chemistry, Carbon neutral laboratory, University of Nottingham NG7 2GA
Email: madasamy.thangamuthu1@nottingham.ac.uk, andrei.khlobystov@nottingham.ac.uk

Supplementary figures

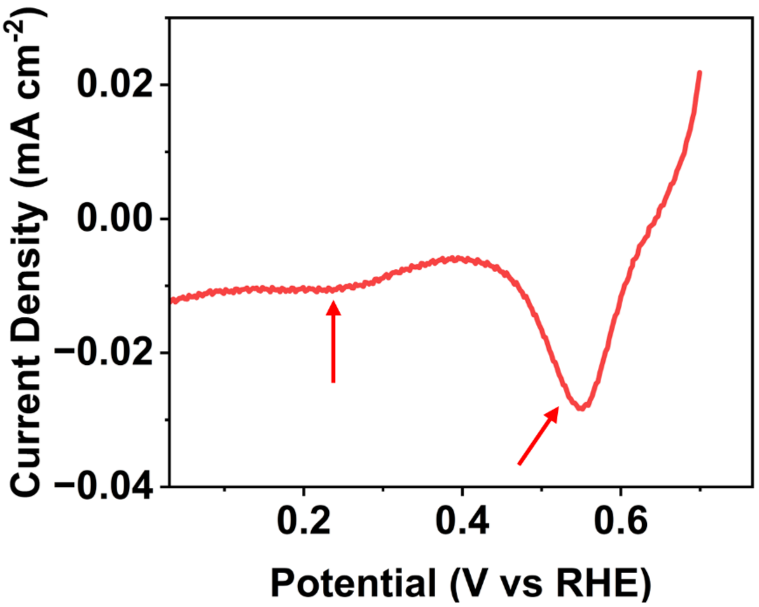

Figure S1. LSV of as-sputtered Cu/GNF at a scan rate of 10 mV s^{-1} measured in 0.1 M $KHCO_3$, illustrating two copper reduction peaks.

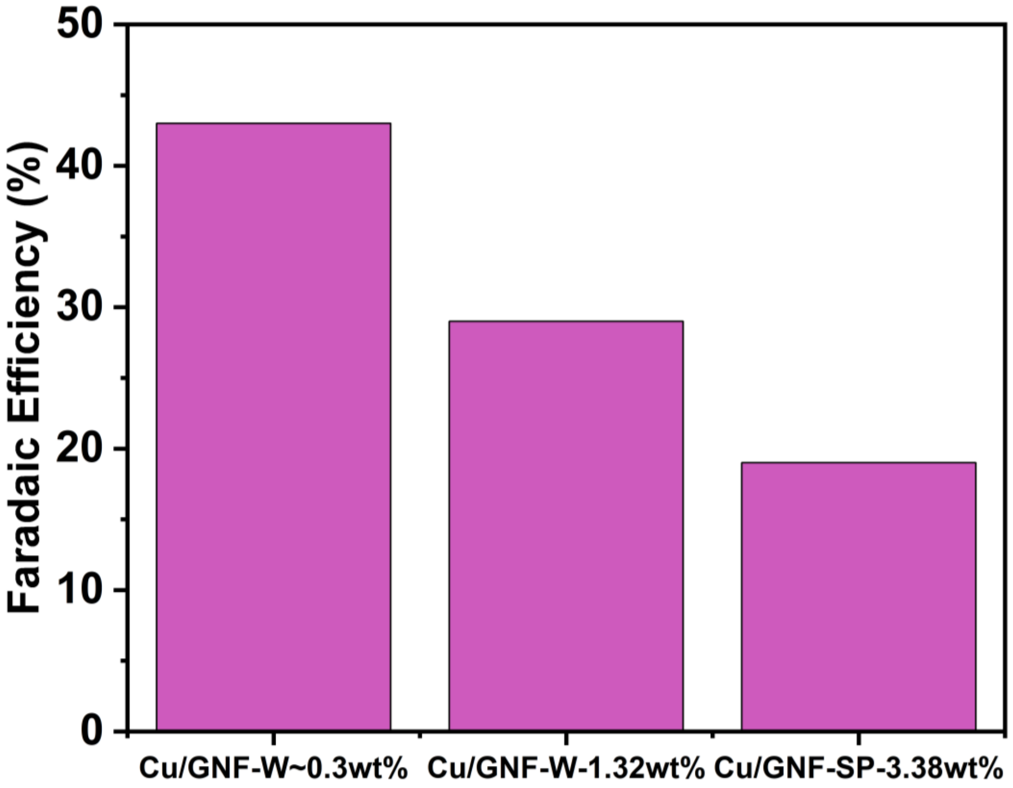

Figure S2. FE of formate at -0.38 V vs RHE obtained for wet chemistry prepared Cu/GNF catalysts and higher weight loading of the sputtered sample. (W represents wet chemistry prepared and SP sputter prepared).

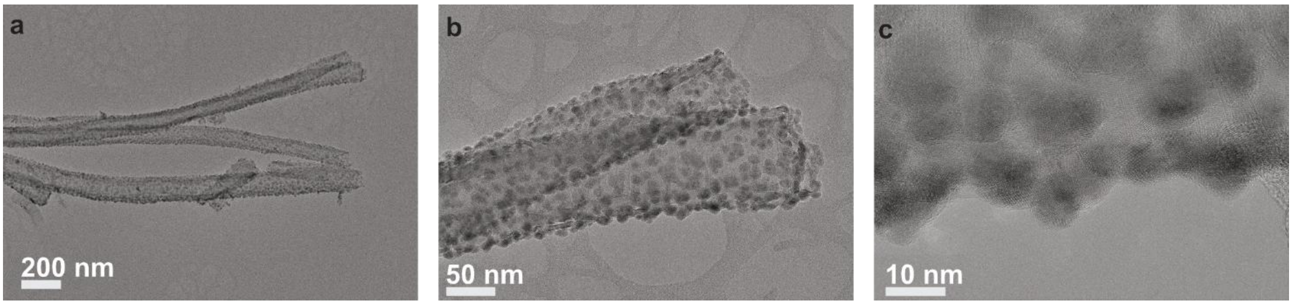

Figure S3. a-c) HR-TEM of 3.38 wt.% weight loading of Cu on GNF

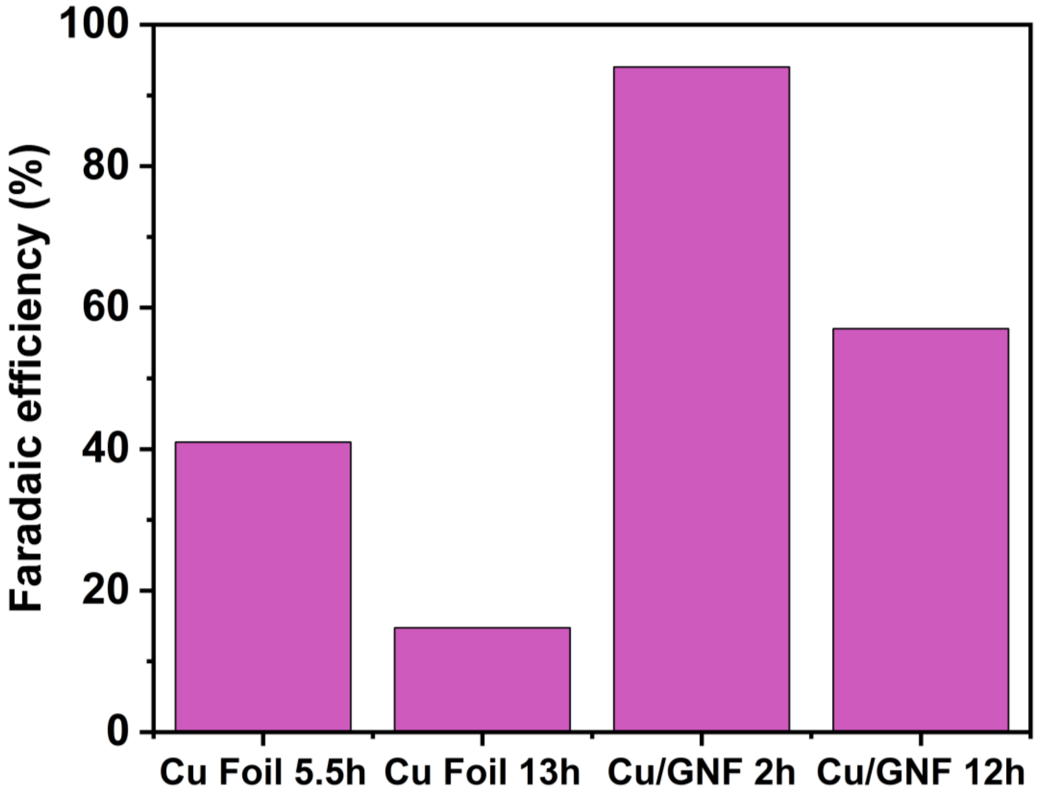

Figure S4. FE of formate of Cu foil after 5.5 h and 13 h vs. GNF/Cu after 2 h and 12 h at -0.38V vs RHE

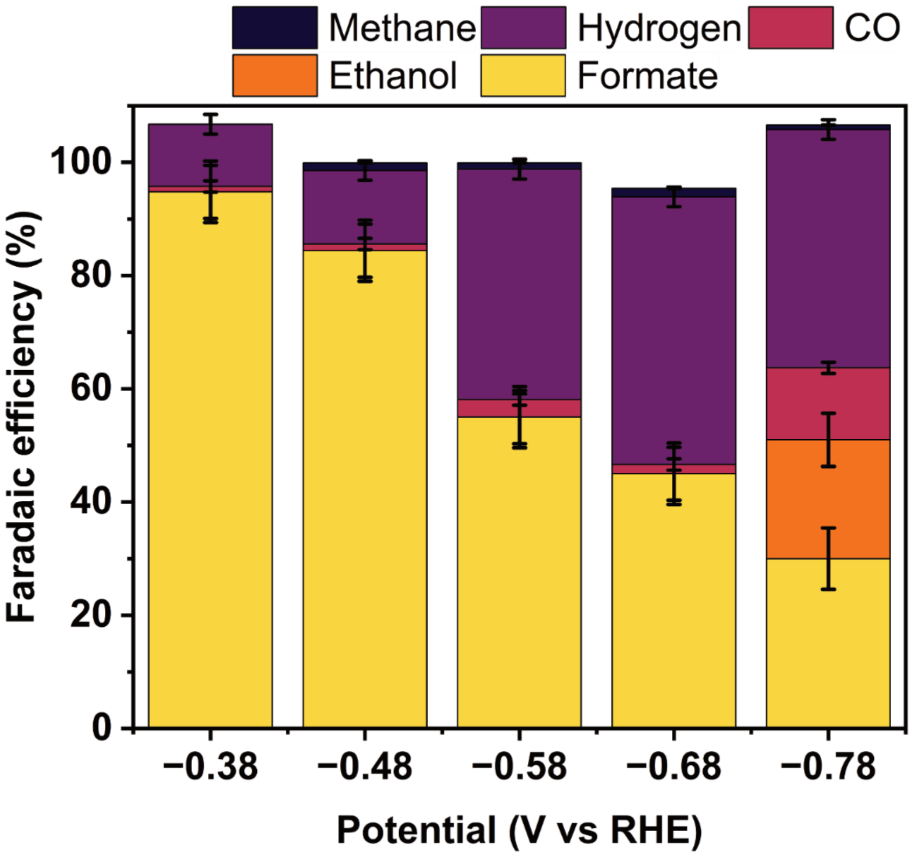

Figure S5. Faradaic efficiency including detectable gas products from -0.38 to -0.78 V vs RHE.

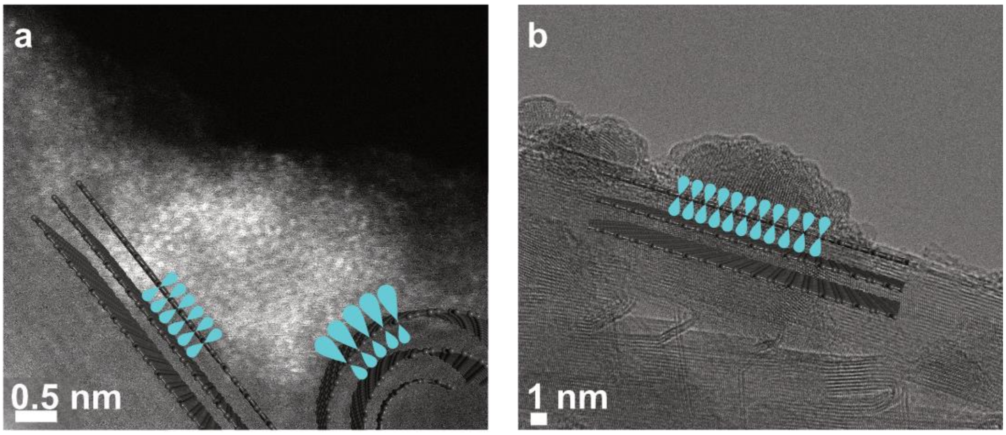

Figure S6. Schematic representation of the carbon π orbital interactions with surface Cu on PR-24 (with external step edges (a) and PR-19 (without external step edges) GNFs (b)

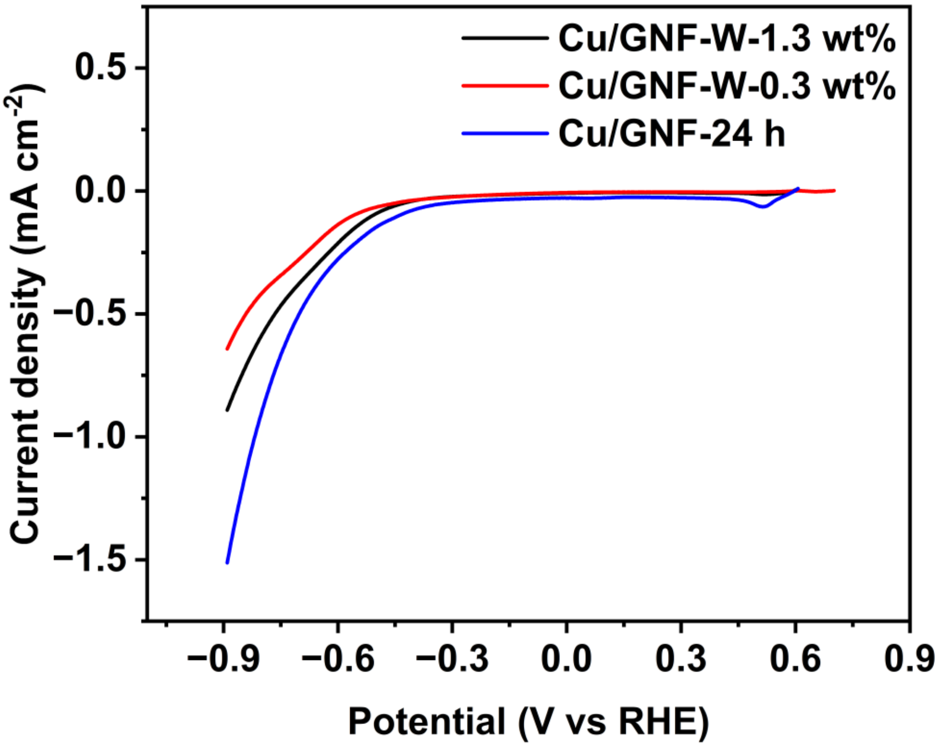

Figure S7. LSV of wet chemistry prepared Cu/GNF measured in 0.1 M $KHCO_3$ sweeping from +0.6 to -0.88 V vs RHE at a scan rate of 10 mV s^{-1}, where W denotes wet chemistry prepared.

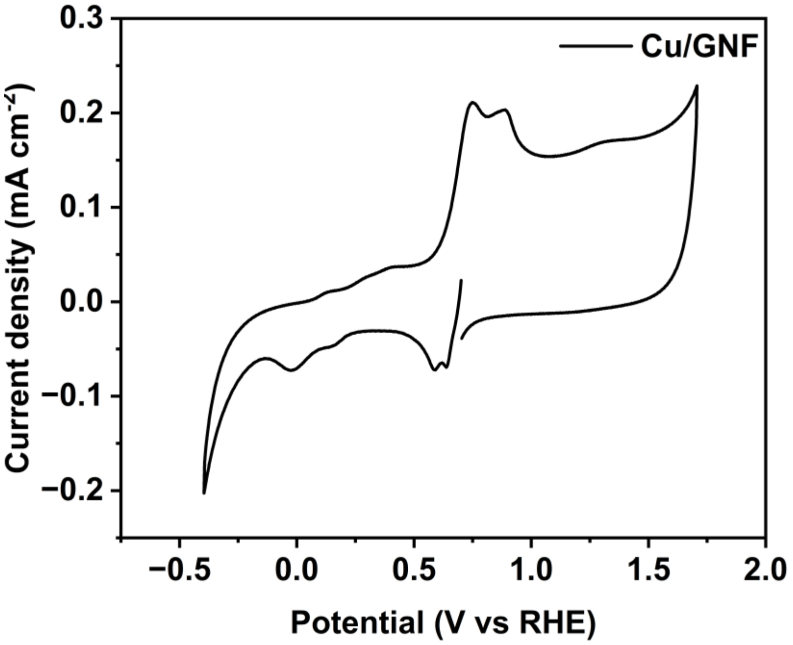

Figure S8. Post-reaction (24 h) CV of Cu/GNF from 1.8 to -0.48V vs RHE at a scan rate of 10 mV/s in reaction electrolyte (0.1 M $KHCO_3$).

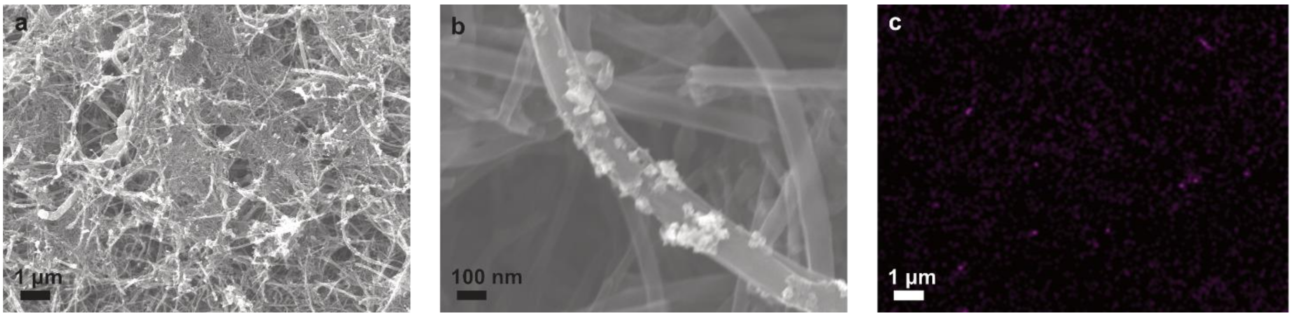

Figure S9. a and b) SEM of the used catalyst after 24 hours at -0.37V vs RHE. c) EDX-mapping of Cu of a).

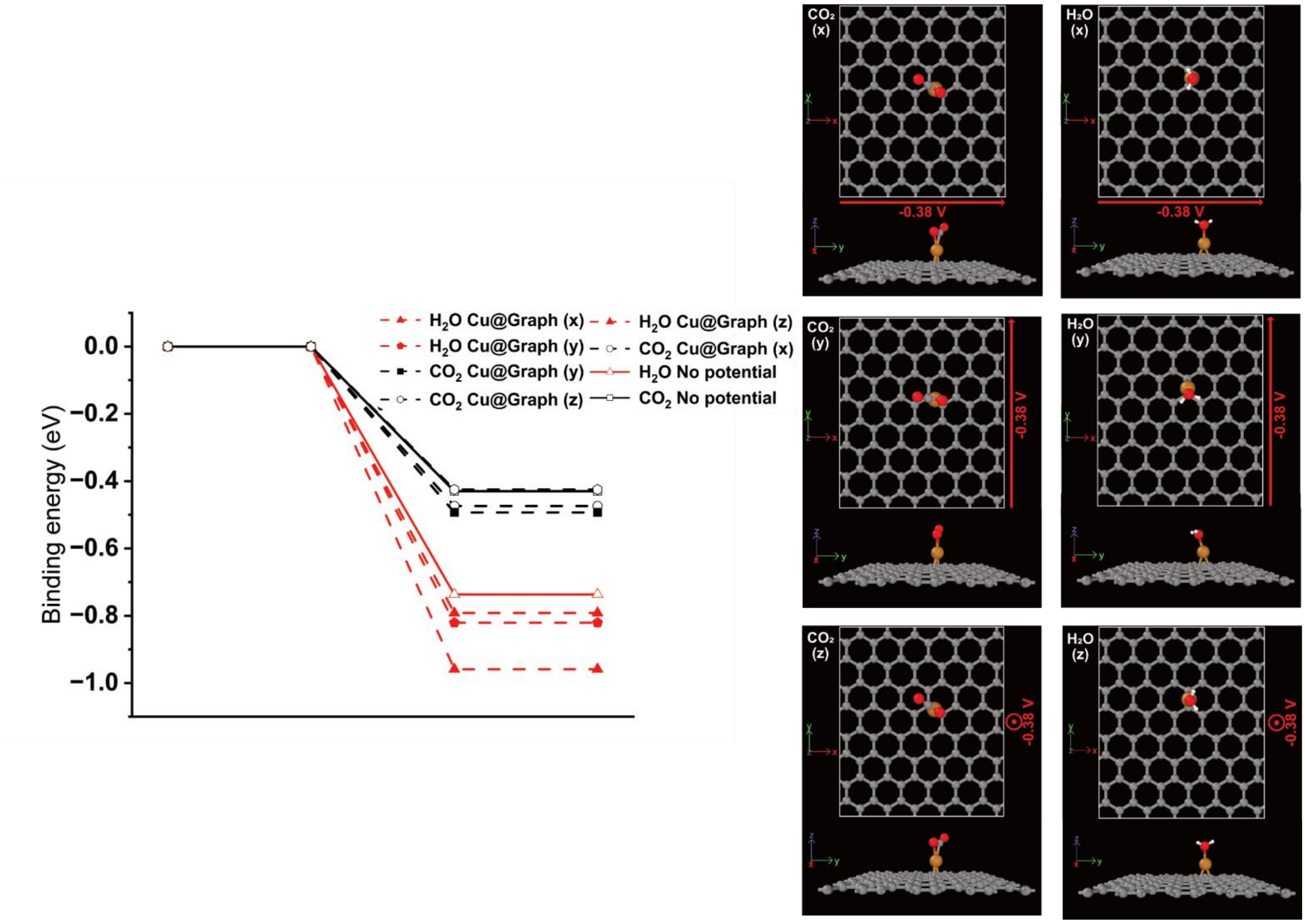

Figure S10. The binding energy of CO_2 and H_2O on Cu atom adsorbed on graphene under external applied filed of -0.38 V along the X, Y and Z-axis.

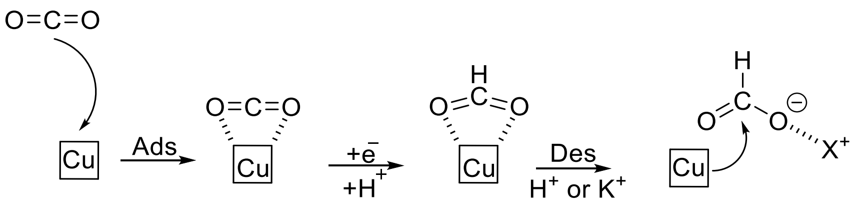

Figure S11. Proposed mechanisms(s) for formate on NCs of Cu

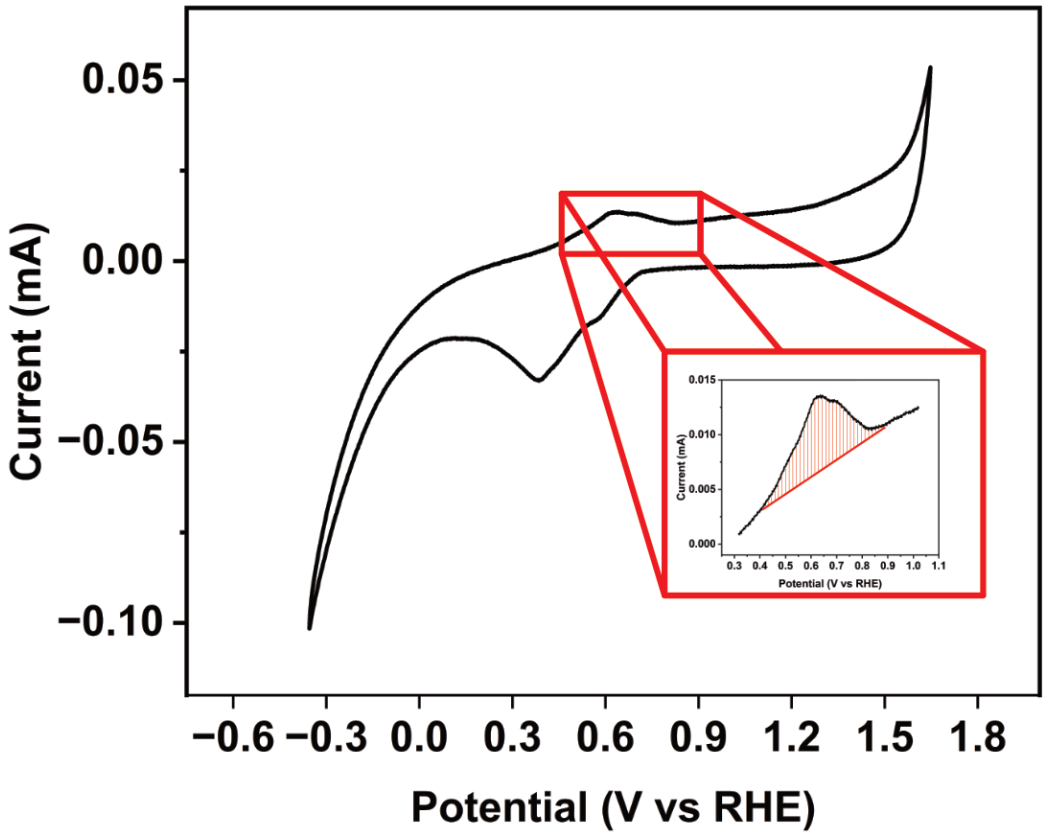

Figure S12. Integration of Cu oxidation on the anodic sweep of a CV sweeping at 10 mV/s, in 0.1 M $KHCO_3$ from -0.36 V to 1.65 V vs RHE.

Table S1. Comparison of PR-19 (no step-edges) and PR-24 GNFs FE for formate

Catalyst	Potential (V vs RHE)	Faradaic efficiency (%)
(PR-19) GNF with no step-edges	-1.18	1.03
	-0.88	3.12
	-0.38	5.43
(PR-24) GNF with step-edges	-1.18	10.5
	-0.88	22.8
	-0.38	94.8

Table S2. Cu $p_{3/2}$ XPS results for Cu/GNF materials, including % peak area.

Sample	Oxidation State	Binding E nergy (eV)	Peak Area	% Peak Area
Cu/GNF	(Cu^0+Cu^I)	932.82	4512	40.8
	Cu^{II}	934.79	4006	36.2
	Sat2	941.83	1537	13.9
	Sat1	944.33	1000	9.1
Cu/GNF-24 h	(Cu^0+Cu^I)	932.86	2062	52.6
	Cu^{II}	934.81	1322	33.7
	Sat2	942.92	503	12.8
	Sat1	944.69	161	0.9

Cu/GNF

(Cu^0+Cu^I) = 33.7 % // (Cu^{II}) = 66.3%

Cu/GNF-24 h

(Cu^0+CuI) = 60.4 % // (Cu^{II}) = 39.6%

Table S3. Comparison of literature Cu-based electrocatalyst with the present Cu/GNF

Catalyst	Year	Electrolyte	Potential (V vs RHE)	FE of formate (%)	Reference
Cu/GNF	2024	0.1 M $KHCO_3$	-0.38	94	This work
Cu/N-Doped porous Carbon	2023	0.1 M $KHCO_3$	-0.70	52	[1]
$Cu/CuO_x/SnO_x$ on porous carbon	2023	0.5 M $KHCO_3$	-1.1	69	[2]
Cu_1Bi_2 Aerogel	2022	0.5 M $KHCO_3$	-0.90	96	[3]
Cu-FTGDE	2024	0.5 M $KHCO_3$	-0.90	76	[4]
Cu_2SnS_3	2023	0.1 M $KHCO_3$	-1.20	92	[5]
SU-101-Cu@2.5C	2023	0.5 M $KHCO_3$	-0.96	95	[6]
Cu/Bi_2S_3-2.67%-N_2	2023	0.5 M $KHCO_3$	-0.80	94	[7]
$Pd_{73}Cu_{27}$	2023	0.5 M $KHCO_3$	-0.56	81	[8]
Cu-Pd/MXene	2023	0.1 M $KHCO_3$	-0.50	79	[9]
Bi_9Cu_1	2023	0.5 M $KHCO_3$	-0.80	98	[10]

Supplementary Note 1

Gas product calculations. Example for H_2

$$FE(\%) = \frac{Q_{product}}{Q_{total}} \times 100 = \frac{n \times F \times f_{gas} \times t \times Product_{moles}}{Q_{total} \times 24.4 \times 10^3} \times 100$$

$$FE = \frac{2 \times 96485\ Cmol^{-1} \times 5\ mlmin^{-1} \times 60\ min}{24.4x10^3\ mL \times 29.69\ C} \times \left(\frac{11.36 \times 10^{-6}\ mol}{0.1\ ml} \times 45\ ml\right) \times 100$$

$$FE = \frac{57891000 \times 5.11x10^{-3}}{7.24x10^5} \times 100$$

$$FE = \frac{2.96x10^5}{7.24x10^5} \times 100 = 40.88\ \%$$

Where n number of electrons for hydrogen generation, F is Faraday constant, f_{gas} is the flow rate of CO_2, t is time of injection, $product_{moles}$ is the amount of moles of product, $24.4x10^3$ is the molar volume of 1 mole of gas and Q_{total} is the charge passed after time t.

Supplementary Note 2

Liquid product calculations. Example for Formate

Concentration in NMR tube:

$$Concentration = 0.333 \times \frac{(0.0228 \times 6)}{(1 \times 3)} = 0.015\ mM$$

Concentration in 0.4 mL aliquot:

$$Concentration = \frac{(0.015 \times 0.000488)}{0.0004} = 0.018\ mM$$

Moles in H-cell:

$$Moles = \frac{(0.018 \times 0.035)}{1000} = 6.48x10^{-7} moles$$

Charge passed to form product:

$$Charge = 6.48x10^{-7} \times 96485.33 \times 2 = 1.25x10^{-1}\ C$$

Faradaic efficiency:

$$FE = \frac{1.25x10^{-1}}{1.32x10^{-1}} \times 100 = 94.7\ \%$$

Supplementary References:

1. Vijayakumar, A. *et al.* A Nitrogen-Doped Porous Carbon Supported Copper Catalyst from a Scalable One-Step Method for Efficient Carbon Dioxide Electroreduction. *ChemElectroChem* **10**, e202200817 (2023).

2. Alkoshab, M. Q. *et al.* Modulating Cu/CuxO Amount in Cu/CuxO–SnOx Nitrogen-Doped Porous Carbon Cuboids toward Low Overpotential CO2 Conversion to Formate. *ACS Appl Energy Mater* **6**, 10794–10806 (2023).

3. Li, H. *et al.* High Performance 3D Self-Supporting Cu−Bi Aerogels for Electrocatalytic Reduction of CO2 to Formate. *ChemSusChem* **15**, e202200226 (2022).

4. Mustafa, A. *et al.* Self-supported copper-based gas diffusion electrodes improve the local CO2 concentration for efficient electrochemical CO2 reduction. *Front Chem Sci Eng* **18**, 29 (2024).

5. Liu, Y. *et al.* Ligand-Controlled Electroreduction of CO2 to Formate over Facet-Defined Bimetallic Sulfide Nanoplates. *Nano Lett* **23**, 5911–5918 (2023).

6. Zou, Y.-H., Wang, X., Ning, F., Yi, J. & Liu, Y. Implanting MWCNTs in BiCu-MOFs to enhance electrocatalytic CO2 reduction to formate. *Sep Purif Technol* **317**, 123806 (2023).

7. Tian, M. *et al.* Doping and pretreatment optimized the adsorption of *OCHO on bismuth for the electrocatalytic reduction of CO2 to formate. *Nanoscale* **15**, 4477–4487 (2023).

8. Todoroki, N., Ishijima, M., Cuya Huaman, J. L., Tanaka, Y. & Balachandran, J. Composition sensitive selectivity and activity of electrochemical carbon dioxide reduction on Pd–Cu solid-solution alloy nanoparticles. *Catal Sci Technol* **13**, 5025–5032 (2023).

9. Abdinejad, M. *et al.* Insertion of MXene-Based Materials into Cu–Pd 3D Aerogels for Electroreduction of CO2 to Formate. *Adv Energy Mater* **13**, 2300402 (2023).

10. Wu, W., Zhu, J., Tong, Y., Xiang, S. & Chen, P. Electronic structural engineering of bimetallic Bi-Cu alloying nanosheet for highly efficient CO2 electroreduction and Zn-CO2 batteries. *Nano Res* (2023) doi:10.1007/s12274-023-6269-7.
